# The emerging therapeutic target of dynamic and reversible N6-methyladenosine modification during cancer development

**DOI:** 10.3389/fonc.2022.970833

**Published:** 2022-09-26

**Authors:** Shougeng Liu, Sihong Chen, Chengfang Tang, Yingxi Zhao, Wei Cui, Lina Jia, Lihui Wang

**Affiliations:** ^1^ Department of Pharmacology, Shenyang Pharmaceutical University, Shenyang, China; ^2^ Benxi Institute of Pharmaceutical Research, Shenyang Pharmaceutical University, Shenyang, China

**Keywords:** m6A modification, cancer, clinical perspectives, cancer immunity, RNA epigenetics

## Abstract

As a reversible and dynamic epigenetic modification, N6-methyladenosine (m6A) modification is ubiquitous in eukaryotic cells. m6A methylation is prevalent in almost all RNA metabolism processes that affect the fate of cells, including cancer development. As indicated by the available evidence, targeting m6A regulators may play a crucial role in tumor therapy and multidrug resistance. Currently, many questions remain uncovered. Here, we review recent studies on m6A modification in various aspects of tumor progression, tumor immunity, multidrug resistance, and therapeutic targets to provide new insight into the m6A methylation process.

## Introduction

**Figure 1:**
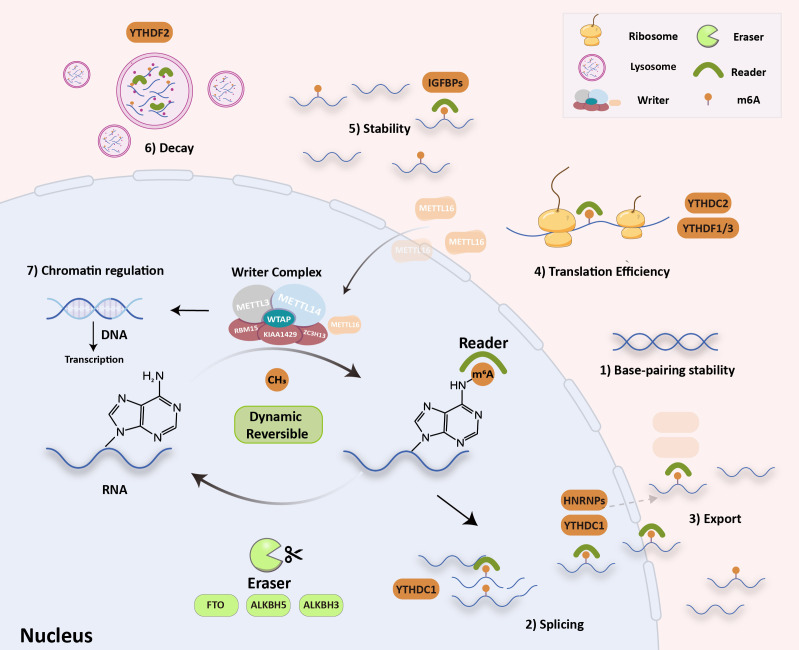
RNA m6A modification molecular mechanism. The writers (METTL3/14, METTL16, WTAP, KIAA1429, RBM15 and ZC3H13) install m6A, the erasers (FTO, ALKBH5/3) remove it, and the readers recognize it (YTH, HNRNPAs, eIF3 and IGF2BP). These can influence: 1) RNA base-pairing stability, 2) RNA splicing, 3) RNA export, 4) translation efficiency, 5) RNA stability, 6) RNA decay process and 7) chromatin regulation.

The N6-methyladenosine (m6A) modification is the most abundant internal modification of RNA in eukaryotic cells, and it has been found in nearly all cellular transcripts. In recent years, high-throughput sequencing technology has become more sensitive and in-depth, which has resulted in the rapid growth of epigenetics. Some research has shown that the m6A modification could affect the stability of RNA base pairing and could control how RNA is spliced, exported, translated, kept stable, and decayed (**Figure 1**) (1–3).

The m6A modification is a dynamic and reversible process that is regulated by m6A “writers” (m6A methyltransferases), m6A “erasers” (m6A demethylases), and m6A “readers” (m6A binding proteins). “Writers” are composed of methyltransferase complexes that were first partially purified from the extracts of HeLa cell nuclei in 1994 (4). The writer complex is a type of heterodimer composed mainly of two methyltransferases, methyltransferase-like 3 (METTL3) and methyltransferase-like 14 (METTL14). The methyltransferase-associated ligands include METTL3 adapter Wilms tumor 1-associated protein (WTAP), KIAA1429, RNA-binding motif protein 15 (RBM15), and zinc finger CCCH domain-containing protein 13 (ZC3H13). METTL3 and METTL14 play the most essential regulatory roles in m6A modification and act as the METTL3–METTL14 complex. By identifying methylation sites, they can regulate accuracy post-transcriptionally. Some studies have noted that METTL3–METTL14 could assist writers in enhancing methylation efficiency with high precision (5, 6). In addition to the above-listed writers, METTL16, a new m6A writer, was recently discovered. Interestingly, METTL16 is preferentially enriched in the cytoplasm, which differs slightly from the localization of METTL3–METTL14 complexes (**Figure 1**) and enters the nucleus to function as a methyltransferase. It controls methionine adenosyltransferase 2A (MAT2A) intron retention (IR) in response to intracellular S-adenosyl-L-methionine (SAM) levels, especially in nascent RNA transcripts. Studies have shown that induction of MAT2A splicing requires METTL16 binding but not methyltransferase activity. Therefore, unlike conventional m6A methylases, METTL16 serves as both an m6A writer and reader (7). “Erasers” can selectively remove methylation of m6A in RNA, which is mediated by fat mass and obesity-associated protein (FTO), alpha-ketoglutarate-dependent dioxygenase alkB homolog 5, 3 (ALKBH5 and ALKBH3) (8, 9). The epigenetic changes in m6A methylation and demethylation suggest that m6A modification in RNA is a dynamic and reversible process (2). “Readers” can specifically decode m6A-modified targeted RNA for the m6A group to trigger biological functions, in which several special proteins are involved, including YT521-B homology (YTH), heterogeneous nuclear ribonucleoproteins (HNRNPs), eukaryotic initiation factor 3 (eIF3), and insulin-like growth factor 2 mRNA-binding proteins (IGF2BPs). Methylation of RNA m6A is a labeling process that needs to be read by m6A “reader” proteins. Marked RNA by m6A methylation could recruit m6A reader proteins that recognize and bind to various m6A sites and trigger different RNA processing destinies (10–12). Recent discoveries have suggested that m6A modification also regulates chromatin. Endogenous retroviruses (ERVs) account for 10% of mammalian genes, and intracisternal A-particles (IAPEz, IAPs) is a type of ERV that is highly active in rodents. IAPs provide functional initiation elements and control the local epigenetic landscape through changes in DNA methylation and histone (protein 3 lysine 9, H3K9) modification. This changes the transcriptional profile of neighborhood genes. Xu et al. proposed that when METTL3 binds to ERV on the transposon subpopulation of IAPs, it causes heterochromatin and represses IAPEz elements from being transcribed, which results in the restriction of ERVs (13). The H3K9 trimethyltransferase SET domain bifurcated histone lysine methyltransferase 1 (SETDB1) and its cofactor tripartite motif containing 28 (TRIM28) can bind to and silence IAPEz (14). This process depends on YTH domain-containing 1 (YTHDC1) and facilitates METTL3-chromatin binding. YTHDC1 is required to maintain mouse embryonic stem cells (EC) identity and retrotransposon repression by recognizing a subset of m6A-marked transposable elements-derived transcripts and then recruiting SETDB1 (15, 16). Specifically, SETDB1-mediated H3K9me3 is dependent on YTHDC1 and METTL3. These findings have shown that changing m6A is a previously undefined key role in chromatin remodelling. In general, m6A is involved in almost all RNA metabolism processes. These alterations eventually participate in different cellular biological processes, such as signaling pathway transduction, cell malignancy biology, hematopoietic function, and body immunity (16). Much evidence has proved that m6A modification participates in tumor malignant progression, oncogenic protein expression, multidrug resistance, and immune microenvironment. Therefore, it is crucial to clarify the role of m6A in cancer pathogenesis and progression. This review summarizes recent studies on m6A modifications in tumor immunity, drug resistance, and targeted therapy to provide new insights into the m6A modification process.

## Abnormal regulation of m6A modification in tumor progression

Dysregulated m6A is closely related to promoting and/or suppressing cancer, affecting cancer progression and patient prognosis. In different cancers, m6A modification displays potential effects on the occurrence and development of cancer (**Table 1**) (50). Therefore, finding the abnormal expression of m6A regulators and figuring out how they work at the molecular level are beneficial in predicting cancer diagnosis and finding valuable treatment targets.

**Table 1:** The role of m6A modifications in cancers.

m6A regulator	Function	Indications	Target	References
METTL3	Oncogenes	Acute myeloid leukemia	*c-MYC, BCL2, PTEN*	(17)
		Hepatocellular carcinoma	*SOCS2*	(18)
		Colorectal Carcinoma	*SRY-box2, MYC, SOCS, CCNE1* (cyclin E1)	(19)
		Retinoblastoma	PI3K/AKT/mTOR signaling pathway	(20)
METTL14	Oncogenes	Acute myeloid leukemia	*MYB, MYC*	(21)
	Suppressor	Hlioblastoma	*ADAM19* (ADAM metallopeptidase domain 19)	(22)
		Hepatocellular carcinoma	microRNA 126	(23)
		Colorectal cancer	LncRNA *XIST*	(24)
METTL16	Oncogenes	Hepatocellular carcinoma		(25)
WTAP	Oncogenes	Leukemia	Hsp90	(26)
KIAA1429	Oncogenes	Hepatocellular carcinoma	*GATA3* (GATA binding Protein 3)	(27)
RBM15	Oncogenes	Head and neck tumors	*TMBIM6* (transmembrane BAX inhibitor motif containing 6)	(28)
ZC3H13	Oncogenes	Colorectal cancer	RAS-ERKsignaling pathway	(29)
FTO	Oncogenes	Acute myeloid leukemia	*ASB2*, *RARA*	(30, 31)
		Breast cancer	BNIP3	(32)
ALKBH5	Oncogenes	Acute myeloid leukemia	*TACC3, AXL*	(33, 34)
YTHDF2	Oncogenes	Acute myeloid leukemia	*Tal1* (TAL BHLH transcription factor-1, erythroid differentiation factor)	(35)
		Bladder cancer	*SETD7, KLF7*	(36)
	Suppressor	Hepatocellular carcinoma	*IL11, SERPINE2*	(37)
YTHDF1	Oncogenes	Colorectal cancer	*ARHGEF2*	(38)
YTHDF3	Oncogenes	Breast cancer	*ST6GALNAC5* (ST6 N-Acetylgalactosaminide Alpha-2,6-Sialyltransferase 5), *GJA1* (gapjunction protein alpha 1), *EGFR* (epidermal growth factor receptor), *VEGFA* (vascular endothelial growth factor A)	(39).
YTHDC1	Oncogenes	Acute myeloid leukemia	*MCM4*	(40)
YTHDC2	Suppressor	Lung adenocarcinoma	*SLC7A11* (solute carrier family 7 member 11)	(41)
YTHDC2	Oncogenes	Hepatocellular carcinoma		(42)
IGF2BP1	Oncogenes	Leukemia	*HOXB4* (homeobox B4), *MYB* (MYB proto-Oncogene, transcription factor), *ALDH1A1* (aldehyde dehydrogenase 1 family member A1)	(43)
		Rndometrial cancer	*PEG10* (paternally expressed 10)	(44)
		Bladder cancer	*MYC*, *FSCN1* (fascin actin-bundling protein 1)	(45)
		Lung adenocarcinoma	*CTNNB1* (catenin beta 1)	(46)
		Glioblastoma	*MYCN* (MYCN proto-oncogene, BHLH transcription factor)	(47)
IGF2BP3	Oncogenes	MLL-Af4 leukemia	miR-873	(48)
IGF2BP2	Oncogenes	Colorectal cancer	*MYC*	(49)

### Writer

In 2017, METTL3, encoding the major m6A methyltransferase, was identified as an essential oncogene in acute myeloid leukemia (AML) (17). A more in-depth study conducted in the same year found that METTL3 was significantly more abundant in AML and promoted the mRNA translation of c-MYC (proto-oncogene), BCL2 (BCL2 apoptosis regulator), and phosphatase and tensin homolog (PTEN) (51). More subsequent studies have also demonstrated that METTL3 is significantly upregulated in hepatocellular carcinoma (HCC) (20), colorectal cancer (CRC) (18), retinoblastoma (RB) (20), and multiple solid tumors. Overexpression of METTL3 significantly promoted HCC progression through the posttranscriptional silencing of cytokine signaling 2 (SOCS2). This process is dependent on the m6A reader YTH N6-methyladenosine RNA-binding protein 2 (YTHDF2) (18). The “writer” protein METTL3 can methylate and stabilize SRY sex-determining region Y (SRY)-box 2 (SOX2) mRNA, which promotes the invasion and migration of colorectal cancer cells. This process requires the involvement of the specific m6A reader insulin-like growth factor 2 mRNA-binding protein 2 (IGF2BP2) (19). METTL3 also promotes the malignant proliferation of CRC cells by stabilizing m6A-modified oncogenic mRNAs (such as MYC proto-oncogene protein, MYC) or by accelerating the decay of suppressor mRNAs (such as SOCS2) (20, 52, 53). As a carcinogenic gene, upregulation of METTL3 promotes the occurrence and development of RB through the PI3K/AKT/mTOR signaling pathway (20). METTL14, another “writer” protein, usually works in combination with METTL3 as a dimeric form, which is required to stabilize the METTL3 conformation and facilitate RNA-binding. Studies have shown that METTL14 is also overexpressed and oncogenic in the development of AML (21), similar to METTL3. However, some research has pointed out that METTL13 and METTL14 have diametrically opposite effects. In cases of glioblastoma (GBM) (22), HCC (23), and CRC (24), METTL14 is identified as a tumor suppressor and downregulated. Downregulating METTL14 decreases m6A levels and increases HCC metastatic capacity. Loss of METTL14 in CRC cells results in decreased m6A-methylation levels of long noncoding RNAs (LncRNA) X-inactive specific transcript (XIST). This results in a reduction of the YTHDF2-m6A-dependent RNA degradation process, leading to elevated LncRNA XIST levels and promoting the tumorigenicity and metastasis of CRC (24). This peculiar performance suggests that the dimer form does not mean that they will always work together. This difference may be attributed to the fact that METTL3 works independently of METTL14 on mRNA translation in the cytoplasm, whereas METTL3 exerts this role in the nucleus through interaction with METTL14 (54). The selective processing of the dimer and the different downstream signals may lead to this seemingly contradictory difference (24). Significantly, METTL3 can solely regulate the m6A modification in endometrioid epithelial ovarian cancer (EOC) (55). Furthermore, Choe et al. found that METTL3 can also stimulate ribosome translation through its interaction with eIF3h (eukaryotic translation initiation factor 3 subunit H) and regulate cancer-related gene translation and tumor progression. Based on the above, it seems that METTL3 can cause a cancer-related function without methyltransferase activity (56). One possible reason is that METTL3 plays a reader role in some types of cancer, which is independent of methyltransferase activity (57). Therefore, targeting METTL3 alone should consider the results of the action triggered by METTL14, and the interaction between METTL14 and METTL3 is also a potential target for cancer therapy. In addition to the METTL14–METTL3 complex, METTL16 has been discovered more recently, and its regulatory role in cancer has attracted some discussion. Su et al. found that METTL16 plays a procarcinogenic role in HCC, and that eukaryotic translation initiation factor 3 subunit A/B (eIF3a/b) shares potential targets with approximately 50% of METTL16’s targets, indicating methyltransferase structural domain of eIF3a/b as a valuable therapeutic target in cancer treatment (25).

The remaining parts of the writer’s complex were also involved in various cancer processes. WTAP, a novel client protein of cell proliferation-related protein (Hsp90), plays an important role in abnormal leukemia cell proliferation and differentiation (26). KIAA1429 is capable of recruiting and directing catalytic core methyltransferase components to specific RNA regions for m6A methylation. Tian et al. proposed that the high expression of KIAA1429 is associated with a poor prognosis in HCC patients. Silencing KIAA1429 inhibits cell proliferation and metastasis in vitro and in vivo (27). RBM15 is significantly elevated in head and neck tumors (LSCC) and correlates with a poor prognosis (28). ZC3H13, which acts as an oncogene in CRC, synergistically inactivates the RAS–ERK signaling pathway, and inhibits cell proliferation and invasion (29).

### Eraser

FTO is the first m6A-modified demethylase discovered that affects mRNA expression and stability. In 2011, Professor Chuan of the University of Chicago demonstrated for the first time that FTO is a demethylase of m6A (8). This discovery attracted significant attention and sparked a wave of RNA epigenetics research. In 2017, FTO was first discovered to play an oncogenic role in AML (30, 31, 58). Li et al. indicated that FTO is abnormally upregulated in AML subtypes with t (11q23)/MLL-rearranged (Mixed Lineage Leukemia gene), t (15, 50), FLT3–ITD (fms-related receptor tyrosine kinase 3) and/or NPM1-mutated (nucleophosmin 1). FTO can reduce the m6A levels of two key target gene mRNAs: ankyrinrepeat and SOCS box containing 2 (ASB2) and retinoic acid receptor a (RARA). This is a demethylation modification in the untranslated region (UTRs) independent of YTHDF2 and YTHDF3, which causes a decrease in mRNA stability and downregulates the expression of related proteins. This provides the possibility that there exist other uncharacterized readers or other regulators with uncharacterized roles.

Recent independent studies have found that there is no significant correlation between the expression levels of METTL3, METTL14, WTAP, FTO, and YTHDF2, and the prognosis of AML. However, the increased expression of ALKBH5 is related to the poor prognosis of AML patients (31, 33, 50), which is due to increased leukemic stem cells’/leukemiainitiating cells’ (LSCs/LICs) self-renewal. Some studies have identified transforming acidic coil containing protein 3 (TACC3) and AXL receptor tyrosine kinase as important ALKBH5 targets. ALKBH5 regulates mRNA stability in an m6A-dependent manner. Shen et al. proposed that targeting ALKBH5 and/or TACC3 chimeras with potent small-molecule inhibitors specifically degrade their proteins (e.g., proteolytic targeting agents) (33, 34). Overall, we suggest ALKBH5 as a viable therapeutic target for AML without affecting normal hematopoiesis. In breast cancer, upregulation of FTO downregulates the m6A epigenetic modification and decreases the expression of BCL2-interacting protein 3 (BNIP3), which is dependent on YTHDF2, a proapoptotic protein. Downregulation of YTHDF2 plays an important role in expansion of hematopoietic stem and progenitor cells (HSPC) and LSCs/LICs function (59–61), by leading to significant promotion of cell proliferation, colony formation, and reduction of apoptosis (32), which suggests it as a viable new target for AML therapy (35). A similar study in breast cancer has shown that decreased METTL3 expression is accompanied by increased ALKBH5 expression, which significantly decreases m6A levels in immune cells, resulting in oncogenic transformation (62).

### Reader

The m6A readers containing the YTH structural domain are well-studied readers that affect multiple steps of RNA metabolism. YTH family proteins participate in almost all processes of cancer malignancy and play an oncogenic role in most cancer types. YTHDF2 is the first reported m6A reader. YTHDF2 and METTL3 were upregulated in bladder cancer (BCA). METTL3-catalyzed m6A modifications of the SET domain-containing 7 (SETD7) and kruppel-like factor 7 (KLF7) mRNAs, which function as tumor suppressors, are recognized by YTHDF2 for mRNA decay. Thus, the signaling axis composed of METTL3/YTHDF2-SETD7/KLF4 is a potential therapeutic target for BCA (36). Hou et al. found that YTHDF2 suppressed tumor cells and tumor vasculature by processing interleukin 11 (IL11) and serpin family E member 2 (SERPINE2) mRNA decay (37). YTHDF1, which has been recognized as an oncogene in many studies, can promote the translational expression of several genes that influence the fate of tumors. The cancer genome atlas (TCGA) database is also the only protein of the YTH family with higher-than-normal colorectal cancer (CRC) expression (50). High expression of YTHDF1 is associated with a poor prognosis in CRC patients. Nishizawa et al. indicated that the oncogenic gene c-MYC can activate the expression of YTHDF1, although the specific downstream signaling effects have not been elaborated. However, they also demonstrated that such activation does not affect other YTH family members (63). In 2022, Wang’s team used YTHDF1 knockdown mice and primary CRC organoids to demonstrate that YTHDF1 can upregulate rho guanine nucleotide exchange factor 2 (ARHGEF2) gene expression and promote CRC metastasis and genesis (38). This study supplements the work of Nishizawa et al. on YTHDF1 downstream-related pathways.

YTHDF3, YTHDC1, and YTHDC2 are less important to cancer than YTHDF1 and YTHDF2. Chang et al. found that YTHDF3 is upregulated in 33% of breast cancer brain metastases and can promote translation by binding to its own mRNA (39). YTHDC1 is the only nucleus-localized protein of the YTH family and is highly expressed in AML of many different nuclear forms. This process is regulated by minichromosome maintenance complex component 4 (MCM), a critical regulator of DNA replication (40). YTHDC2 has been suggested to be associated with lung cancer and HCC, but relevant details remain uncovered (41, 42). Interestingly, recent studies have shown that YTHDC2 is associated with mammalian meiotic progression, which may be a promising direction for further exploration (18).

In February 2018, IGF2BP wasfirst identified as one of the m6A “readers” camp. It is important for cell growth, development, differentiation, and stem cell maintenance. IGF2BPs promote the stability and dependence of their target mRNAs (e.g., MYC) in m6A in a manner that affects gene expression output (64). According to Lederere et al., IGF2BP1 and IGF2BP3 are, however, true fetal proteins. De novo synthesis is significantly upregulated in the tumor group, which increases the aggressiveness of tumor cells (65). IGF2BP1 plays a dominant role as an m6A reader and is involved in a variety of cancer processes, such as leukemia (43), endometrial cancer (EC) (44), BCA (45), lung adenocarcinoma (LUAD) (46), and glioblastoma (GBM) (47). IGF2BP1 promotes an aggressive tumor phenotype primarily by impairing the miRNA protection of its effector mRNAs. For example, upregulation of IGF2BP1 reverses the inhibitory effect of miR-873 on GBM cell progression (48). Mentioning IGF2BP3 has led to a recent discovery. Tran et al. found that deleting IGF2BP3 in MLL-Af4 leukemia can decrease cancer aggressiveness, delay cancer progression, and increase the survival rate of MLL-Af4 leukemia mice (49). It has been further determined that IGF2BP3 is required for efficient initiation of MLL-Af4 leukemia and LIC function. IGF2BP2 is the only family member commonly expressed in adult mouse tumors and is engaged in the collective metabolic processes of cancer. Wang et al. found that long intergenic noncoding RNA for IGF2BP2 stability (LINRIS) has a positive interaction with IGF2BP2. It can bind to the ubiquitination site of IGF2BP2 and block the degradation of IGF2BP2 via the ubiquitination–autophagy pathway (66). First, this study elucidates the degradation pathway of IGF2BP family members. The interaction between IGF2BP2 and LINRIS regulates the rate of glycolysis by regulating the expression of MYC mRNA and its downstream. LINRIS knockdown downregulates MYC-related metabolic enzymes and value-added blockage in cancer cells. All these experimental results confirm the drug-forming therapeutic potential of targeting the LINRIS-IGF2BP2-MYC axis (66).

## Aberrant regulation of m6A modification in cancer immunization

Following the in-depth study of tumor immunity, the role of m6A-mediated epigenetic modification in the tumor immune system or immune microenvironment (TIME) has gained increasingly more attention and studies. Recent research reported that m6A-modified mRNA can affect the differentiation and proliferation of T cells, which constitute the basis of adaptive immunity. The m6A modification is closely related to the most promising immunotherapies, such as anti-PD-1 (program cell death ligand 1)/PD-L1 (programmed death ligand 1) monotherapy, cancer vaccines, and big data virtual screening. As a result, m6A modulators are important for screening suitable immunotherapy candidates to achieve precision medicine. Although whether the RNA epitranscriptome is involved in immune evasion remains unknown, these findings collectively suggest that m6A-targeted therapies combined with PD-1/PD-L1 ICIs (immune checkpoint inhibitors) could improve the effect of tumor immunotherapy.

METTL3 can promote the methylation of SOCS family member mRNA, resulting in an increase in its translation level, which negatively regulates the signal transduction of IL-7 in CD4+ T cells and blocks the proliferation of T cells (67). Knockdown of METTL14 and YTHDF1 could reduce mRNA transcript levels of interferon signaling IFNA/IFNB/IFNG/ISG5, which is involved in the formation of the gastric cancer (GC) microenvironment and suppresses the antitumor immunity mediated by interferon signaling blocking (68, 69). The depletion of methyltransferase METTL3 or METTL14 increases the sensitivity of CRC cells to interferon gamma (IFN-γ). This type of downregulated m6A modification can increase the transcripts of the IFN-ß-Stat1-Irf1 axis and induce mRNA stabilization mediated by the m6A reader YTHDF2. This suggests that a methyltransferase inhibitor/anti-PD-1+anti-CTLA-4 (cytotoxic T-lymphocyte-associated antigen 4) intervention can be used to inhibit tumor growth (70).

**Figure 2:**
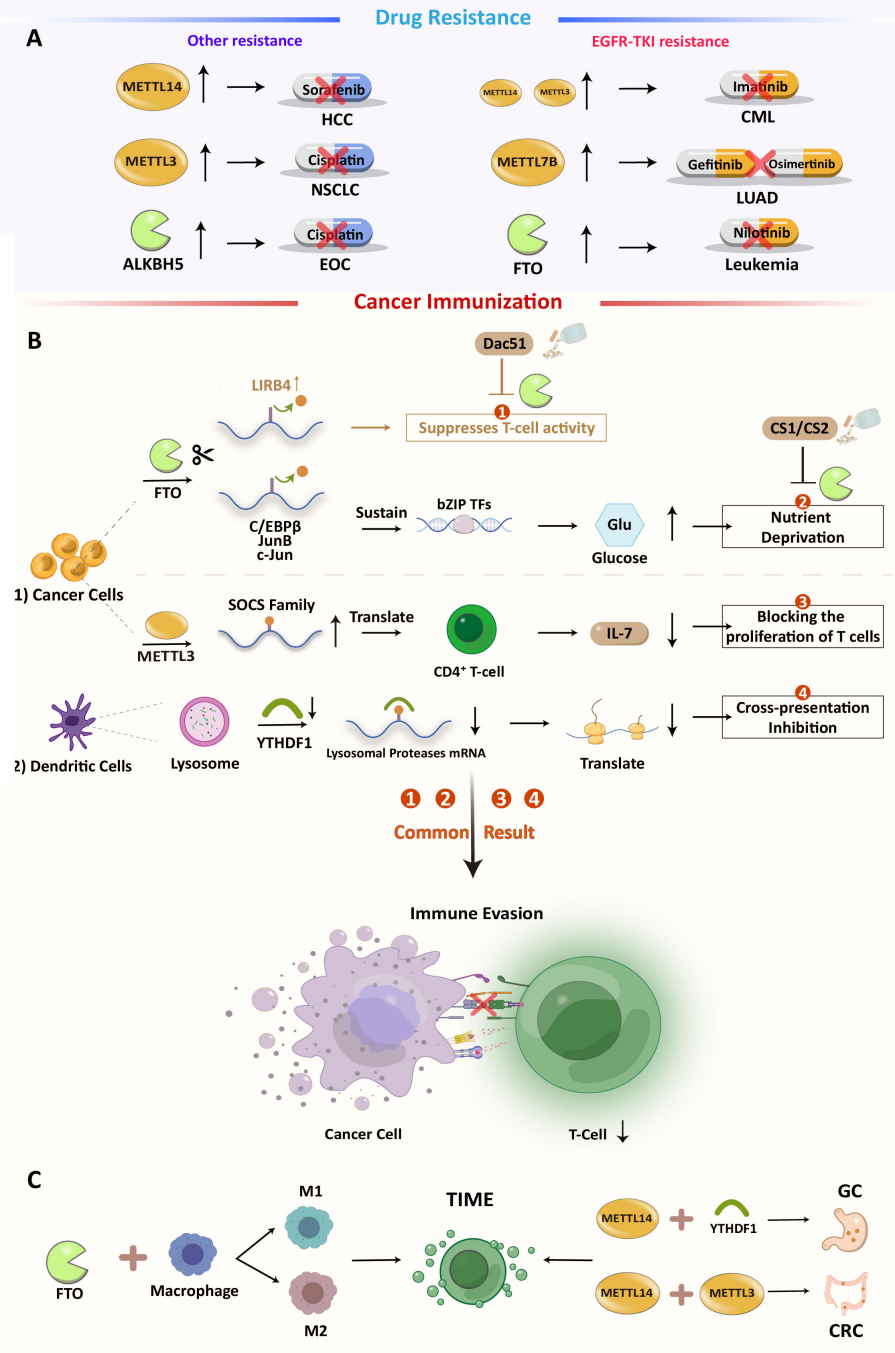
**(A)** Overview of m6A-mediated antitumor drug resistance **(B)** m6A modification regulates immune in cancers and the possibility of inhibiting Dac51 or YTHDF1 with ICIs:1). FTO-mediated direct inhibition of T cells. 2). FTO induces nutrient deprivation. 3). METTL3 promotes the expression of SOCS family mRNA and inhibits T cell proliferation. 4). Methylation of related protease mRNA m6A in DC lysosomes triggers inhibition of cross-presentation. **(C)** Some m6A regulators can also have an effect on TIME.

According to a study, FTO can preserve the basic leucine zipper (bZIP) family of an oncogenic transcription factor to dispose of the expression of glycolytic-associated protein and increase glycolysis. In addition, FTO loss can increase CD8+ T cell infiltration and cytotoxicity. In summary, as an immunosuppressive molecule, FTO may limit T cell activation and effector status in multiple tumors. Furthermore, a potential FTO inhibitor, Dac51, has been discovered. It can abolish the effects of FTO and restore T cell responses, suggesting an FTO inhibitor as a combination therapy for PD-1 therapy (**Figure 2B**) (71). In TIME, macrophages play an important role in initiating immune responses. Macrophages infiltrating tumor tissue become tumor-associated macrophages (TAM). Previous research has suggested that macrophages can clear cancer cells. However, with advanced research, TAM has been proven to promote tumor immune escape. The main polarization types of TAMs are M1 and M2. M1-type TAMs have tumor-killing effects, while M2-type TAMs promote tumor progression, invasion, and migration. The imbalance between M1 and M2 TAMs is a characteristic of various diseases (72, 73). Different forms of polarization are regulated by different transcription factors. For example, the signal transducer and activator of transcription (STAT1) and hypoxia-inducible factor 1-alpha (HIF-1α) promote M1 polarization, while STAT3, STAT6, and peroxisome proliferator-activated receptor gamma (PPAR-γ) promote the M2 type. However, the suppressor of cytokine signaling 3 (SOCS3) can suppress the pro-inflammatory phenotype of M1. Gu et al. found that the expression of FTO decreased M1 and M2 polarization by inhibiting the polarization process. They also found that the expressions of STAT1, STAT6, and PPAR-γ were positively correlated with FTO. In the FTO knockdown model, the m6A levels of STAT1 and PPAR-γ mRNAs were increased, which is dependent on YTHDF2 recognition, resulting in decreased mRNA stability and abundance. FTO also inhibits the NF-κB signaling pathway (72). Together, these results demonstrate that FTO has the ability to promote polarization in the TAM. The regulation of macrophage polarization may become a new target for immunotherapy. In primary AML, leukocyte immunoglobulin-like receptor B4 (LILRB4) is highly expressed (its expression level is 40–50 times higher than that of PD-L1 and PD-L2 in AML) and promotes tumor infiltration through suppressing T cell activity and triggering immune evasion beyond PD-L1 and L2. FTO can upregulate LILRB4 through an m6A-mediated modification process, and FTO inhibitors can effectively reduce their expression and activate T cells. Moreover, in the treatment of elderly AML patients with hypomethylating agents (HMG), upregulation of immune checkpoint gene expression often occurs, resulting in immune escape, and drug resistance. Therefore, the combination of the FTO inhibitor and HMA (5-azacytidine and decitabine) may be a better therapeutic strategy for AML treatment (74, 75).

YTHDF1 recognizes and activates the translation of lysosomal cathepsins in dendritic cells. Loss of YTHDF1 in classical dendritic cells increases cross-presentation of tumor antigens and antigen-specific CD8+ T cell antitumor responses, through boosting PD-L1 checkpoint blockade’s therapeutic efficacy. YTHDF1 has been recognized as a potential anticancer immunotherapy target (76). Li et al. found that knocking down ALKBH5 decreased Mct4 (monocarboxylate transporter 4)/Slc16a3 (solute carrier family 16 member 3) mRNA and cancer cell lactate concentrations. Decreased lactate concentration in the TME is associated with impaired myeloid-derived suppressor cells (MDSC) and regulatory T cell (Treg) expansion and differentiation. Therefore, ALKBH5 may also be a possible therapeutic target for treating cancer, alone, or with immune checkpoint blockade (ICB) (77). In prostate cancer (PCA), Liu et al. constructed an m6A score system to quantify m6A in individual PCA patients. The system is based on multidimensional cluster analysis of m6A regulatory clusters, immune clusters, and co-expression genes (CEGs) of 24 m6A regulators’ modification patterns (78). This system demonstrated that three modes of m6A regulation define the transcriptomic and immune-infiltrating signatures of PCA (low, medium, and high). It can reflect m6A modification and the prognosis of PCA. A similar scoring model was constructed in CRC to quantify the RNA modification-related subtypes of CRC and the RNA modification “writer” score (WM_score), which is based on the mRNA expression pattern of 26 RNA modification “writers”. In this assessment method, two distinct RNA modification patterns were identified and characterized by a high and a low WM_Score. Tumors in the low WM_score group are sensitive to ICB therapy and demonstrate better prognostic outcomes, while tumors with a high WM_score maintain tumor immune tolerance. The abundance of immune cells in the tumor microenvironment differs significantly between these two CRC subtypes, and the high WM_score subtype is associated with a higher infiltration of suppressive immune cells, including M2 macrophages, plasma cells, tregs, and Tfh (T follicular helper cells) cells (79). These bioinformatics methods are beneficial for understanding m6A modification in TME. All these analyses suggest that RNA modification patterns are a good “predictor” of chemotherapy or immunotherapy outcomes.

Numerous recent studies have concluded that the m6A-mediated RNA epitranscriptome is engaged in the process of tumor immune evasion, which can facilitate the development of a new class of epitranscriptome targets for immunotherapy. The m6A modification is strongly associated with ICI resistance in cancer. Therefore, targeting the involvement of immune cells and other stromal cells in TIME remodeling, in addition to targeting specific m6A sites, is a promising strategy for the treatment of TIME. There is still hope for the future, despite the fact that combinatorial strategies for m6A-mediated immunotherapy still require extensive research.

## Aberrant regulation of m6A modification in drug resistance

Both genetic and epigenetic alterations cause anticancer drug resistance, but only the latter can be reversed (80, 81). As shown in **Figure 2A**, some studies have suggested that m6A modifications are engaged in cancer progression and drug resistance (**Figure 2A**).

In HCC, METTL14 methylated and turned down hepatocyte nuclear factor 3 (HNF3). This is linked to the cancerous nature of the tumor, a bad prognosis, and resistance to sorafenib. Significantly, IGF2BPs are involved in the stabilization of HNF3 mRNA (82), which may serve as potential therapeutic targets for reversing this resistance pathway. METTL3 overexpression can directly induce non-small cell lung cancer (NSCLC) cisplatin (DDP) resistance by promoting YAP (Yes1-associated transcriptional regulator) translation with high mRNA methylation (83). Furthermore, METLL3 also participates in exosomal miR-4443-induced DDP resistance by inhibiting FSP1- (ferroptosis suppressor protein 1)-mediated ferroptosis (84, 85). Aberrant regulation of m6A modification affects lung cancer prognosis and afatinib resistance (86). In LUAD, METTL7B is a potential therapeutic target for reversing gefitinib- and osimertinib-resistance by promoting three key reactive oxygen species scavengers (ROS) (87, 88).The dysregulation of the FTO-m6A axis in leukemia cells is required for EGFR-TKI (tyrosine kinase inhibitors) resistance, which can be prevented or eradicated by specifically targeting FTO (89). In chronic myelogenous leukemia (CML), METTL3, and METTL14 are upregulated and affect sensitivity to TKI imatinib (90). In EOC, another demethylase, ALKBH5, maintains JAK2 (Janus kinase 2) mRNA expression by abrogating JAK2 mRNA methylation and reducing YTHDF2-mediated mRNA degradation. This results in resistance to cisplatin by activating the JAK2/STAT3 signaling pathway through the ALKBH5-HOXA10 cycle (91).

Multiple lines of evidence, as mentioned previously, suggest that targeting m6A modification can sensitize cancer cells to antitumor agents in a variety of cancer types. The combination of m6A-targeted drugs and chemotherapy or targeted molecules may provide novel approaches for overcoming drug resistance. As an innovative area of tumor resistance or recurrence, m6A epigenetic modification is anticipated as a biomarker and therapeutic target in malignant tumors.

## Promising therapeutic targets in m6A modification for cancer therapy

RNA m6A modification is a potential therapeutic target in many cancers. Moreover, the expression of some m6A-modified regulators in normal human tissues is lower than in cancers (74). These findings suggest that targeting the main regulatory regulators of m6A is a promising therapeutic target for cancer therapy without serious adverse reactions. In this section, we decided to mainly summarize and discuss relevant cutting-edge research on m6A agonists or inhibitors (**Table 2**).

**Table 2:** RNA m6A Modified agonists or inhibitors Discussion and future perspectives.

m6A regulator target	Compound	Indication	Function	References
m6A writer complex	Piperidine derivative and piperazine derivative compounds		METTL3-enzyme complex activator	(92)
METTL3	STM2457	Leukemia	1). enhances the expression of CD40^+^ 2). works on crucial stem cell lines and promotes phenotypicreversal	(93)
FTO	Rhein		competitively binds the catalytic domain of FTO	(94)
FTO	MA		competes for m6A-containing substrate binding with ssDNA	(95)
FTO	R-2HG	Leukemia, Glioma	targets the FTO/m6A/MYC/CEBPA axis to trigger an increase in global mA levels	(96)
FTO	FB23-2	Acute myeloid leukemia	inhibits the LSCs of AML by inhibiting FTO and its downstream targets such as *MYC*, *CEBPA*,*RARA* and *ASB2* RNA transcripts	(97)
FTO	CS1, CS2	Acute myeloid leukemia	1). occupies FTO catalytic pocket to reduce the demethylase activity of FTO 2). decreases self-refinement capacity of LSCs/LICs,	(98)
				
				
FTO	Dac51		inhibit the effects of FTO and restores T-cell responses	(71)
ALKBH5	Compound 3, compound 6	Glioblastoma, acute myeloid leukemia	1). regulates mRNA exon splicing ratio and self-expression in melanoma 2). regulates the content of metabolites and cytokines in tumor microenvironment	(99)
IGF2BP1	Herb Tripterygium wilfordii Hook	Nasopharyngeal carcinoma	downregulates IGF2BP1 mRNA targets	(100)
IGF2BP1	Compound 7773	Lung adenocarcinoma	binds to IGFBP1 and inhibits its ability to conjunct with *Kras* mRNA	(101)
IGF2BP1	BTYNB	Melanoma,Ovarian cancer	prevents IGF2BP1 from binding to *c-MYC* mRNA and inhibits cell proliferation	(102)

### Writer

The core part of m6A “writers” consists of METTL3-METTL14 and WTAP methyltransferase complex. Selberg et al. used advanced virtual screening techniques and selected SAM as the binding site of the METTL3-METTL14 complex. They also found two representative small molecules: Ligands (tumor-specific small-molecule ligands) compounds 1 (piperidine derivative) and 4 (piperazine derivative) (92). Unfortunately, their potential anticancer effects have not been verified. STM2457, a bioavailable and highly selective METTL3 inhibitor, can significantly increase CD40+ expression, reduce the presence of AML cells from peripheral blood, act on crucial stem cell lines, and promote phenotypic reversal to prevent the development of AML. This was the first demonstration of the antitumor efficacy of m6A “writer” inhibitors (93).

### Eraser

FTO belongs to the superfamily of non-heme Fe (II)/2-oxoglutarate (2OG)-dependent dioxygenases. Recent studies have associated m6A dysregulation with myeloid malignancies, providing new insights into AML pathogenesis and drug resistance (59). In 2012, Rhein, a natural product, was identified by structure-based computer screening as the first competitive inhibitor that competitively binds the catalytic domain of FTO (94). Subsequently, they also found that meclofamic acid (MA) was identified as a selective inhibitor of FTO without affecting other eraser proteins, such as ALKBH5. MA can inhibit FTO demethylation efficiently and selectively by competing for m6A-containing substrate binding with ssDNA (95). With the in-depth study of a-KG and MA, more efficient FTO inhibitors have been developed. In the treatment of AML, R-2HG (produced by mutated isocitrate dehydrogenase 1/2 (IDH1/2) catalyzes the conversion of α-KG), an oncometabolite, inhibits the enzymatic activity of FTO and displays a significant antitumor effect in leukemia and glioma by targeting the FTO/MYC/CEBPA (CCAAT enhancer binding protein alpha) axis (59, 103, 104). This inhibition triggers an increase in global m6A levels, including the accumulation of m6A on MYC-encoding RNA, resulting in a decrease in MYC signaling. R-2G can also improve the effect of first-line chemotherapeutic drugs, such as HMA, on AML cells. However, such antitumor effects have certain limitations: They appear to be difficult to apply clinically due to the limited activities, and they are only feasible in 2-HG-sensitive and wild-type IDH AML cells. IDH-mutated AML patients are mostly 2-HG resistant. When the IDH gene is mutated, its catalytic product, R-2HG, can competitively inhibit the demethylase activity of FTO. In addition, the overactivation of MYC induces the resistance of IDH2-mutated cells to R-2HG. For IDH mutant cancers, IDH inhibitors and FTO inhibitors can be combined to block the recovery of FTO functional activity caused by the reduction of R-2HG. Additionally, they pointed out that JQ1 (MYC signaling pathway inhibition) can inhibit the R-2HG desensitization process caused by MYC enhancement, making it a better combination therapy. In the follow-up study about MA, two derivatives of MA were developed, FB23, and FB23-2, which can inhibit the LSCs of AML in vivo through inhibiting FTO and its downstream targets, such as MYC, CEBPA, RARA, and ASB2 RNA transcripts, and re-enriching m6A. Furthermore, FB23-2 can inhibit the proliferation of AML cell lines and primary AML LSCs in PDX (a patient-derived xenotransplantation) mice (97, 105). In an issue of cancer cell in 2020, Su et al. identified two new potent FTO small-molecule inhibitors, CS1 (NSC337766, Bisantrene) and CS2 (NSC368390, Brequinar), by using structure-based virtual screening (SBVS). These inhibitors could inhibit the MYC pathway and induce cell apoptosis in AML, which is at least 10 times more effective than previously reported FTO inhibitors. They could specifically target FTO, binding to its catalytic pocket, and reducing demethylase activity, resulting in a significant increase in apoptosis and cell cycle arrest (in the G0 phase) in human AML cells. The self-refinement capacity of LSCs/LICs is considered a major cause of treatment failure and relapse in AML. The FTO is considered to play a role in the self-renewal/repopulation of LSC/LIC. Encouragingly, 50 nM CS1 almost completely inhibited the repopulating capacity of AML cells. CS1 and CS2 showed higher anti-leukemic efficacy than FB23-2 in vitro and in vivo. Another “eraser” protein, ALKBH5, has been identified as regulating the mRNA exon splicing ratio and self-expression in melanoma. Research on ALKBH5 inhibitors mainly focuses on GSC and AML (98, 106). In addition, ALKBH5 could regulate the content of metabolites and cytokines in the tumor microenvironment during GVAX (a granulocyte-macrophage colony-stimulating factor (GM-CSF) gene-transfected tumor cell vaccine)/anti-PD-1 therapy. This suggests a new combination therapy prospect for PD-1 therapy (77, 99). Rau et al. used gene-editing technology to construct the RCas9-FTO fusion protein with the specific demethylation of the m6A sequence in RNA, which can effectively target RNA and bind to multiple sgRNAs (small guide RNA), then subsequently remove specific (single or multiple) m6A methylations. The construction of this technology makes it possible to pass the introduction/elimination through the m6A site of a specific gene (which can be based on the m6A regulatory element). This precise and effective tool can also explore the combination of different m6A modification effects on cancers (107). The above contents suggest that targeting m6A-related pathways is a worthy therapy for treating AML and even multiple cancers.

### Reader

IGF2BP or YTH domain-containing proteins can enhance mRNA stability and translation by recognizing m6A. After IGF2BP was recognized as an m6A reader in February 2018, relevant research on its inhibitor also gradually emerged (64). For example, in the treatment of human nasopharyngeal carcinoma (NPC), when a conserved but oncogenic LncRNA THOR (testis-associated oncogenic LncRNA, ENSG00000226856) was downregulated, the function of IGF2BP1 was simultaneously inhibited. IGF2BP1 and LncRNA THOR have been proven to have a combinatorial effect. Disruption of Lnc-THOR-IGF2BP1 signaling in NPC cells can effectively inhibit NPC cell growth. Wang et al. extracted a monomer compound from the herb Tripterygium wilfordii Hook, which has been reported to downgrade IGF2BP1 and produce antitumor effects in vivo and in vitro in NPC (100). Wallis et al. found that compound, 7773, binds IGF2BP1and inhibits its ability to conjunct with Kras mRNA, which results in inhibiting cell metastasis and malignant progression of LUAD (101). Another small-molecule inhibitor, BTYNB, has been identified as a potent and selective inhibitor of IGF2BP1. It could inhibit cell proliferation in melanoma and ovarian cancer by interrupting the binding of IGF2BP1 and c-MYC mRNA (102). YTHDF1 is also a novel therapeutic target for cancer immunotherapy (WO2020132536 (A1), from the CDDI database). Targeting YTHDF1 can improve antitumor immune responses by decreasing YTHDF1 activity in immune system cells (e.g., DCs and antigen-presenting cells).

In conclusion, m6A modification persists in numerous cancer types. By regulating the process of epigenetic modification, inhibitors that target m6A modification regulators offer the possibility of intriguing new cancer treatment avenues. However, the evidence presented here suggests that m6A modulator inhibitors are more in line with the concept of precision medicine; additional research is still required.

## Discussion and future perspectives

In summary, m6A modification is closely associated with cancer occurrence, metastasis, tumor self-renewal capacity, cancer metabolic reprogramming, drug resistance generation, and tumor immune microenvironment. Therefore, m6A is a promising biomarker for early diagnosis, individual detection, and predictive detection. Targeting m6A methylation can sensitize multidrug-resistant cancer cells to chemotherapeutic agents or boost the immunotherapeutic efficacy of anti-PD-1 therapy. The investigation of the underlying mechanism of m6A modification focuses on the interplay between various biological processes (e.g., autophagy and ferroptosis), metabolites (e.g., lactate, glucose, and cystine), and ncRNAs (e.g., LncRNA, CircRNA, and miRNA) (50, 96). In fact, we should acknowledge the following facts: 1) Different m6A modifications may be independent of one another, as demonstrated by the opposing effects of METTL3 and METTL14 or between readers and writers of the same type of cancer. 2) In thousands of mammalian transcriptomes, it is difficult to correlate methylation sites with cancer malignancy phenotypes. 3) It needs to be investigated whether m6A modifications of various RNA types interact with one another. 4) M6A modifications in some types of RNA are difficult to detect. Deciphering the effect of m6A modifications on alternative transcripts can aid in the comprehension of the molecular machinery underlying therapeutic resistance and provide a novel strategy for the diagnosis and treatment of cancer (108).

Epigenetic modification of m6A is regarded as a novel target for the development of novel anticancer agents. With the discovery of numerous m6A modification inhibitors and activators in recent years, it will be essential to develop a potential technology for m6A therapy. Currently, research is being conducted on an increasing number of m6A-modified drugs, but there are still issues with side effects and poor specificity. The discovery of m6A-targeted anticancer drugs can be divided into three stages (1): traditional medicine-based natural products (109), such as herb tripterygium wilfordii hook (107) (2), modern chemical modification or synthesis, and (3) artificial intelligence (AI)-assisted approaches (110). Currently, many bioinformatics methods and tools can be used to explore and predict cancer-related information. For example, RNAMethyPro, a comprehensive pan-cancer analysis, consists of seven m6A regulators, which can identify the prognosis of high-risk cancer patients and predict survival at the pan-cancer level (111). Another similar tool is the m6ASNP, dedicated to predicting m6A-disrupting variants, which can reveal the relationship between the m6A modification and other posttranscriptional regulations (112). m6A Atlas (www.xjtlu.edu.cn/biologicalsciences/atlas) is a comprehensive database of the m6A epitranscriptome that displays high-confidence results based on a collection of reliable m6A sites and condition-specific quantitative profiles (113). M6A2 Target is the first database containing the corresponding target genes of three types of m6A methylases (writers, erasers, and readers), and is used to gather information from the published m6A WER-related data for target gene prediction (114). These developments in artificial intelligence technology in the field of pharmacy may make it easier to comprehend abnormal m6A modifications and influencing factors between various m6A modifications and accelerate the research progress of targeting specific m6A modifications.

## Author contributions

Concept and design: LW and LJ. Acquisition of data: SL and CT. Analysis and interpretation: SL and SC. Original Draft: SL, LJ, YZ, and LW. Review and editing: LW and WC. All authors contributed to the article and approved the submitted version.

## Funding

This work was supported by the National Natural Science Foundation of China (No. 82073320, 81903112), the Central Guidance on Local Science and Technology Development Fund of Liaoning Province (nos. 2022JH6/100100038) and the “Xingliao Talents” Program of Liaoning Province (nos. XLYC1902008).

## Acknowledgments

This is a short text to acknowledge the contributions of specific colleagues, institutions, or agencies that aided the efforts of the authors.

## Conflict of interest

The authors declare that the research was conducted in the absence of any commercial or financial relationships that could be construed as a potential conflict of interest.

## Publisher’s note

All claims expressed in this article are solely those of the authors and do not necessarily represent those of their affiliated organizations, or those of the publisher, the editors and the reviewers. Any product that may be evaluated in this article, or claim that may be made by its manufacturer, is not guaranteed or endorsed by the publisher.
